# Assessment of clinical readiness, knowledge and attitude, regarding Basic Life Support (BLS) and cardiopulmonary resuscitation (CPR) skill among dentists practicing in Saudi Arabia

**DOI:** 10.7717/peerj.21098

**Published:** 2026-05-06

**Authors:** Osama Khattak, Aliya Ehsan, Ayesha Akbar Khalid, Azhar Iqbal, Farooq Ahmad Chaudhary, Muhammad Nadeem Baig, Thani Alsharari, Muhammad Rizwan Memon

**Affiliations:** 1 Department of Restorative Dentistry, College of Dentistry, Jouf University, Sakaka, Saudi Arabia; 2 Queen Elizabeth Hospital, England, United Kingdom; 3 School of Dentistry, Shaheed Zulfiqar Ali Bhutto Medical University, Islamabad, Pakistan; 4 Department of Preventive Dentistry, College of Dentistry, Jouf University, Sakaka, Saudi Arabia; 5 Department of Restorative Dental Science, Faculty of Dentistry, Taif University, Taif, Saudi Arabia; 6 Department of Prosthetics, College of Dentistry, Jouf University, Sakaka, Saudi Arabia

**Keywords:** Basic life support, Cardiopulmonary resuscitation, Dentists, Knowledge, Attitude, Clinical readiness, Saudi Arabia

## Abstract

**Background:**

Medical emergencies in dental settings can arise unexpectedly and demand prompt, skilled management. Dentists are often the first responders; therefore, competence in Basic Life Support (BLS) and cardiopulmonary resuscitation (CPR) is essential. This study aims to evaluate the knowledge, attitudes, and perceived structural clinical readiness regarding BLS and CPR among dentists practicing in Saudi Arabia and predictors of good knowledge and confidence in performing CPR.

**Methods:**

A cross-sectional study was conducted among 400 licensed dentists from public and private sectors in Saudi Arabia. Data were collected through a validated, self-administered online questionnaire comprising four sections: sociodemographic characteristics, knowledge, attitudes, and clinical readiness. Binary logistic regression analyses identified predictors of good knowledge and confidence in performing CPR, adjusting for age, gender, designation, experience, workshop attendance, and previous emergency encounters.

**Results:**

Overall, 26.0% of participants demonstrated good knowledge of BLS/CPR, 41.0% had moderate knowledge, and 33.0% had low knowledge 26.0% demonstrated good knowledge. Attendance at a BLS/CPR workshop within the last five years (OR = 4.91; 95% CI [2.60–9.24]; *p* < 0.001) and prior emergency encounters (OR = 12.97; 95% CI [6.99–24.06]; *p* < 0.001) were strong predictors of good knowledge. Confidence in performing CPR was associated with recent workshop attendance (OR = 6.93; 95% CI [4.19–11.46]; *p* < 0.001), while male gender showed lower confidence (OR = 0.59; 95% CI [0.37–0.93]; *p* = 0.023). Only 44.5% reported clinic-level emergency protocols, and 7.5% had an automated external defibrillator.

**Conclusion:**

Dentists in Saudi Arabia exhibit positive attitudes but insufficient knowledge and confidence regarding BLS/CPR. Regular refresher training, simulation-based education, and enforcement of clinic-level emergency preparedness are essential to enhance patient safety and professional competence.

## Introduction

Medical emergencies, though relatively rare in dental practice, can occur unexpectedly and require immediate and competent management to prevent serious complications or fatalities (Laurent et al., 2014). Dentists are often the first and sometimes the only healthcare providers present when such emergencies occur in the dental office. Therefore, having the knowledge, skills, and confidence to initiate Basic Life Support (BLS) and cardiopulmonary resuscitation (CPR) is not only important but an ethical and professional obligation. Dentists, due to the nature of their work and prolonged interaction with patients, often involving sedation, local anesthesia, or stressful procedures are at risk of encountering such emergencies and must be adequately prepared (Zingade, Kumar & Gujjar, 2021).

BLS is a set of basic interventions including chest compressions, rescue breathing, and defibrillation with an automated external defibrillator (AED) (Koster et al., 2010). The American Heart Association (AHA) and other global health bodies emphasize the importance of BLS competency for all healthcare providers. In response, many countries including Saudi Arabia have made BLS training and certification mandatory for licensure and employment in healthcare settings (Abolfotouh et al., 2017; Almukhlifi, Crowfoot & Hutton, 2024). However, evidence suggests that simply holding a certification may not necessarily translate into adequate knowledge or the ability to perform effective CPR in real-life situations (Al Jadidi & Al Jufaili, 2023). Over time, without continuous reinforcement or practical exposure, skills may deteriorate, leading to poor performance in emergencies. Furthermore, several studies conducted globally and regionally have reported deficiencies in knowledge, confidence, and readiness among dentists and other healthcare workers in relation to CPR and emergency management (Al-Iryani et al., 2018; Algarni et al., 2024). These gaps are attributed to factors such as infrequent training, lack of hands-on practice, outdated knowledge, and absence of emergency drills or preparedness protocols in dental clinics (Al-Iryani et al., 2018; Jamalpour, Asadi & Zarei, 2015).

The dental setting in Saudi Arabia is diverse, comprising public sector facilities under the Ministry of Health, academic institutions, military hospitals, and a growing number of private clinics (Walston, Al-Harbi & Al-Omar, 2008). This diversity introduces variations in emergency preparedness protocols, access to equipment like AEDs, and opportunities for skill reinforcement. Despite the existing regulatory framework, there remains a lack of comprehensive national data assessing the actual preparedness of dentists to handle emergencies, particularly their knowledge, clinical readiness, attitudes, and perceived challenges related to BLS and CPR.

Moreover, cultural, institutional, and individual factors may influence a dentist’s response to emergency scenarios. For example, some practitioners may perceive emergencies as low-risk events due to their rarity, while others may feel inadequately supported by their institutions in terms of training or resources (Ng et al., 2023). In addition, recent changes in CPR guidelines, such as the emphasis on compression-only CPR or changes in the compression-to-ventilation ratio, highlight the need for ongoing updates and education among practitioners (Ng et al., 2023).

Understanding the current level of knowledge, preparedness and the challenges faced by dentists is essential for designing targeted interventions. These may include revising curriculum content in dental education, increasing the frequency and quality of BLS training workshops, implementing mock drills, and establishing clinic-level emergency protocols. A proactive approach will not only enhance the confidence and competence of dental professionals but also contribute to improved patient safety and healthcare outcomes. Unlike many previous studies that have focused primarily on assessing knowledge or attitudes toward BLS and CPR among dentists, the present study adopts a more comprehensive approach by simultaneously evaluating knowledge, attitudes, self-perceived clinical readiness, and confidence in managing medical emergencies (Aedh, Abdel-Haliem & Farid, 2025; Albaqami & Abozaid, 2019). In addition, this study examines the availability of emergency resources in dental clinics and identifies independent predictors of good knowledge and confidence using multivariable regression analysis. By providing updated national-level evidence aligned with current CPR guidelines and regulatory requirements in Saudi Arabia. Several previously published instruments have been used to assess BLS and CPR knowledge, skills, and readiness among healthcare professionals (Alkandari, Alyahya & Abdulwahab, 2017; Albaqami & Abozaid, 2019). These tools generally focus on theoretical knowledge of resuscitation guidelines, self-reported confidence, or prior training exposure. However, many of these instruments were developed for specific populations such as medical students, nurses, or general healthcare providers and were primarily based on international guideline frameworks without adaptation to the dental clinical setting (Albaqami & Abozaid, 2019; Dar, Kishk & Nagla, 2025). Furthermore, some tools emphasize knowledge assessment alone and do not comprehensively evaluate practical readiness, perceived barriers, or context-specific challenges faced in dental practice. Given these limitations and the absence of a standardized instrument tailored specifically to dentists within the local context, a study-specific questionnaire was developed to capture clinical readiness, knowledge, attitudes, and perceived challenges relevant to the study population.

The present study aims to assess the clinical readiness, challenges, knowledge, and attitudes regarding BLS and CPR skills among dentists practicing in Saudi Arabia. Specifically, it will explore (1) the level of theoretical knowledge related to BLS and CPR, (2) self-perceived clinical readiness and past experience with emergencies, (3) attitudes toward the importance of CPR in dental settings. By identifying strengths and gaps in current practices, this study seeks to provide evidence-based recommendations to policymakers, dental institutions, and training providers to strengthen emergency preparedness in dental care settings across the Kingdom of Saudi Arabia.

### Methods

### Study design and setting

This cross-sectional study was conducted among licensed dentists practicing in various regions of Saudi Arabia, including both private and government healthcare sectors. Data were collected through a structured, self-administered online questionnaire.

### Study population and eligibility criteria

The study population included general dental practitioners, intern/house officers, faculty specialists, and consultants practicing in Saudi Arabia. Inclusion criteria were: (1) possession of a valid dental license and (2) provision of informed consent to participate. Dentists not currently engaged in clinical practice or those unwilling to participate were excluded.

The questionnaire did not include an item assessing the geographic region of practice; therefore, the regional distribution of participants within Saudi Arabia could not be determined.

### Sample size calculation and sampling technique

The minimum required sample size was calculated using the formula for estimating a single population proportion. Assuming a 50% prevalence of adequate CPR knowledge among dentists (to maximize sample size), a 95% confidence level, and a 5% margin of error, the calculated sample size was 385. To account for potential non-response, the final target sample size was increased to approximately 400 participants.

A convenience sampling strategy was used to recruit participants *via* professional dental associations, social media platforms, and dental clinics. A standardized recruitment message describing the study objectives, eligibility criteria, and voluntary nature of participation was used across all recruitment channels.

### Data collection instrument, its structure and scoring

Data for this study was collected using a structured, self-administered questionnaire developed based on existing literature, BLS/CPR training guidelines, and expert input in dental and emergency care. Content validity was assessed through expert review by a panel comprising senior faculty members in dental public health and emergency care, who evaluated the items for relevance, clarity, and alignment with current clinical practice. The questionnaire was distributed online using survey platforms (*e.g.*, Google Forms), and responses were collected anonymously between September and October 2024. The questionnaire was designed to assess three key domains: knowledge, attitude, and perceived structural clinical readiness, related to BLS and CPR among dentists. The instrument was comprised of four main sections with a total of 27 items. The first section assessed the socio-demographic characteristics of the participants (age, gender, designation, years of experience, encounter emergency in dental practice and attended previous BLS/CPR workshop). Section two, with nine items, assesses participants’ knowledge and understanding of core BLS and CPR concepts through multiple-choice questions (including definitions of BLS and CPR, chest compression rate and depth, compression-to-ventilation ratio, sequence of BLS steps, use of automated external defibrillators (AEDs), recovery position, and rescuer rotation). The items cover definitions, recommended guidelines, and procedural knowledge aligned with international BLS standards (Panchal et al., 2020; Olasveengen et al., 2020). Each correct answer was scored as “1” and incorrect as “0”, with a total possible score of 9. The knowledge level was categorized into Low (1–3), moderate (4–6) and Good (7–9), following a logical division of the total score into lower, middle, and upper thirds. This approach provides a clear and interpretable classification of knowledge levels, allowing for meaningful comparison across participants. The scoring system and cut-off points were adapted from previous literature and reflect the relative adequacy of BLS/CPR knowledge among dentists in the current study (Aedh, Abdel-Haliem & Farid, 2025). In another previous study the participants’ knowledge scores were categorized into poor, moderate, and good based on Bloom’s cut-off points (Good: 80–100%; Moderate: 50–79%; Poor: <50%), which is a commonly referenced approach in health knowledge assessments and aligns with ordinal categorization of total scores into three levels (low/moderate/good) (Dar, Kishk & Nagla, 2025).

Section three is comprised of four items that explores the participants’ attitudes and beliefs regarding the importance and ethical responsibility of performing CPR in clinical settings. Responses are recorded using a dichotomous scale: Agree or Disagree. Attitudinal scores were summarized to assess overall agreement with the importance and relevance of BLS in dental practice.

Section four (eight items) evaluates both the readiness and availability of emergency preparedness measures in the clinic and the self-perceived confidence of dentists in handling emergency situations. Readiness was assessed based on self-reported availability of emergency resources and perceived confidence rather than objective functional assessment. The first four items are answered with Yes/No options and the remaining four items assess self-reported confidence using a 2-point scale: Very confident or Not confident. This section helps determine both the availability of emergency resources and the clinician’s psychological preparedness. This dichotomous scale was selected to simplify response options, reduce respondent burden, and allow clear differentiation between participants who felt adequately prepared and those who did not. While multi-point validated confidence scales may provide greater granularity, the primary objective of this study was to identify whether dentists perceived themselves as sufficiently confident to perform BLS/CPR.

### Pilot testing and reliability

The instrument was pilot-tested on 20 dentists who were not included in the final analysis to assess face validity and comprehensibility. During pilot testing, participants were asked to provide feedback on item clarity, wording, and the overall flow of the questionnaire. Minor modifications were made based on this feedback to improve item clarity and ensure that all questions were easily understood. Internal consistency reliability of the questionnaire domains was assessed using Cronbach’s alpha, which demonstrated acceptable reliability for knowledge (α = 0.78), attitude (α = 0.81), and clinical readiness (α = 0.76).

### Ethical considerations

Ethical approval was obtained from the local committee of bioethics at Jouf University (Ref: 07-06-53) prior to data collection. Informed consent was obtained electronically prior to survey initiation; participants were required to read an information statement and indicate their consent before accessing the questionnaire. Participation was anonymous, and no financial or non-financial incentives were provided. Data was kept confidential and used solely for research purposes.

### Statistical analysis

Data were analyzed using Statistical Package for the Social Sciences **(**SPSS) version 26 (IBM Corp., Armonk, NY, USA). Descriptive statistics (frequencies, percentages, means, and standard deviations) were used to summarize participant characteristics. Associations between demographic variables and outcomes (knowledge, attitudes, and readiness) were examined using chi-square tests, *t*-tests, and Analysis of Variance (ANOVA) as appropriate. A *p*-value < 0.05 was considered statistically significant. Binary logistic regression was performed to identify independent predictors of (i) good knowledge of BLS/CPR (good *vs.* low/moderate) and (ii) confidence in performing CPR (very confident *vs.* not confident). Independent variables entered into both models included age, gender, designation, years of experience, attendance at a BLS/CPR workshop, and prior emergency encounter. Adjusted odds ratios (aOR) with 95% confidence intervals (CI) were reported. Model fit was evaluated using Omnibus *χ*^2^, −2 Log Likelihood, Nagelkerke R^2^, Hosmer–Lemeshow test, and classification accuracy.

## Results

A total of 400 licensed dentists completed the questionnaire and were included in the final analysis. Due to the open online recruitment strategy, the total number of dentists invited to participate could not be determined; therefore, a response rate could not be calculated. Nearly half the participants were aged 21–30 years (48.0%), and the majority were female (55.5%). General dentists (44.5%) and interns/house officers (33.5%) comprised the largest groups. Forty-one percent had 6–10 years of experience. More than half (57.0%) had attended a BLS/CPR workshop within the last 5 years, while only 26.8% reported encountering a medical emergency in practice (Table 1).

**Table 1:** Socio-demographic and professional characteristics of participating dentists in Saudi Arabia (2024) (*n* = 400).

Variables	n (%)
Age	
21–30	192 (48.0)
31–40	146 (36.5)
≥41	62 (15.5)
Gender	
Male	178 (44.5)
Female	222 (55.5)
Designation	
General dentist	178 (44.5)
Intern/house officers	134 (33.5)
Faculty/specialist	88 (22.0)
Years of Experience	
Less than 5 years	136 (34.0)
6–10 years	166 (41.0)
>10	98 (24.5)
Attended BLS/CPR workshop	
Within last 5 years	228 (57.0)
Before 5 years	172 (43.0)
Encounter an emergency in Clinical practice	
Yes	107 (26.8)
No	293 (73.3)

The majority agreed that CPR is critical for dental professionals (85.5%), that they feel morally obligated to help during emergencies (90.5%), and that BLS training should be mandatory for licensure (74.0%). A minority (29.5%) believed BLS was not relevant in dental settings. Attitudes varied significantly by age, gender, designation, and workshop attendance for certain items (*p* < 0.05) (Table 2).

**Table 2:** Attitudes of dentists toward BLS/CPR by participant characteristics.

**Variables**			
	**CPR is a critical skill for all dental professionals** **n (%)**		**I feel morally obligated to help during a medical emergency** **n (%)**		**BLS training should be mandatory for licensure** **n (%)**		**I believe BLS is not relevant in dental settings** **n (%)**	
	Agree 342 (85.5)	Disagree 58 (14.5)	Agree 362 (90.5)	Disagree 38 (9.5)	Agree 296 (74.0)	Disagree 104 (26.0)	Agree 118 (29.5)	Disagree 282 (70.5)
Age								
21–30 31–40 ≥41	150 (78.1) 136 (93.2) 56 (90.3)	42 (21.9) 10 (6.8) 6 (9.7)	164 (85.4) 140 (95.9) 58 (93.5)	28 (14.6) 6 (4.1) 4 (6.5)	134 (69.8) 114 (78.1) 48 (77.4)	58 (30.2) 32 (21.9) 14 (22.6)	68 (35.4) 32 (21.9) 18 (29.0)	124 (64.6) 114 (78.1) 44 (71.0)
*p*-value	0.001		0.003		0.182		0.026	
Gender								
Male Female	146 (82.0) 196 (88.3)	32 (18.0) 26 (11.7)	156 (87.6) 206 (92.8)	22 (12.4) 16 (7.2)	124 (69.7) 172 (77.5)	54 (30.3) 50 (22.5)	64 (36.0) 54 (24.3)	114 (64.0) 168 (75.7)
*p*-value	0.077		0.081		0.077		0.011	
Designation								
General dentist Intern/house officers Faculty/specialist	142 (79.8) 120 (89.6) 80 (90.9)	36 (20.2) 14 (10.4) 8 (9.1)	148 (83.1) 130 (97.0) 84 (95.5)	30 (16.9) 4 (3.0) 4 (4.5)	124 (69.7) 102 (76.1) 70 (79.5)	54 (30.3) 32 (23.9) 18 (20.5)	58 (32.6) 38 (28.4) 22 (25.0)	120 (67.4) 96 (71.6) 66 (75.0)
*p*-value	0.014		0.001		0.177		0.416	
Years of experience								
Less than 5 years 6-10 years >10	108 (79.4) 146 (88.0) 88 (89.8)	28 (20.6) 20 (12.0) 10 (10.2)	116 (85.3) 154 (92.8) 92 (93.9)	20 (14.7) 12 (7.2) 6 (6.1)	98 (72.1) 126 (75.9) 72 (73.5)	38 (27.9) 40 (24.1) 26 (26.5)	44 (32.4) 48 (28.9) 26 (26.5)	92 (67.6) 118 (71.1) 72 (73.5)
*p*-value	0.042		0.037		0.743		0.614	
Attended BLS/CPR workshop								
Within last 5 years Before 5 years	196 (86.0) 146 (84.9)	32 (14.0) 26 (15.1)	208 (91.2) 154 (89.5)	20 (8.8) 18 (10.5)	162 (71.1) 134 (77.9)	66 (28.9) 38 (22.1)	76 (33.3) 42 (24.4)	152 (66.7) 130 (75.6)
*p*-value	0.761		0.567		0.122		0.053	
Encounter an emergency in Clinical practice								
Yes No	88 (82.2) 254 (86.7)	19 (17.8) 39 (13.3)	96 (89.7) 266 (90.8)	11 (10.3) 27 (9.2)	71 (66.4) 225 (76.8)	36 (33.6) 68 (23.2)	43 (40.2) 75 (25.6)	64 (59.8) 218 (74.4)
*p*-value	0.264		0.748		0.035		0.005	

**Notes.**

Chi-square test was used, *p*-value < 0.05 was considered statistically significant

Less than half (44.5%) reported having a BLS/CPR protocol in their clinic, 58.0% had an emergency kit, and only 7.5% reported availability of an AED. While 61.0% expressed interest in refresher training, confidence in skills was low: only 41.0% felt confident in chest compressions, 28.5% in AED use, and 41.5% in recognizing cardiac arrest. Confidence was highest for managing choking (66.0%) (Table 3).

**Table 3:** Clinical readiness and confidence in managing medical emergencies.

**Clinical Readiness & Emergency Response Capacity**	**Yes** **n (%)**	**No** **N (%)**
1.Do you have a BLS/CPR protocol in your clinic?	178 (44.5)	222 (55.5)
2. Is an emergency kit available in your clinic?	232 (58.0)	168 (42.0)
3. Is an AED available in your clinic? Yes / No	30 (7.5)	370 (92.5)
4. Are you interested in attending a BLS/CPR refresher training?	244 (61.0)	156 (39.0)
	**Very confident**	**Not confident**
5. Confidence in performing chest compressions	164 (41.0)	236 (59.0)
6. Confidence in using an AED	114 (28.5)	286 (71.5)
7. Confidence in managing choking	264 (66.0)	136 (34.0)
8. Confidence in recognizing cardiac arrest	166 —(41.5)	234 (58.5)

Overall, 26.0% demonstrated good knowledge, 41.0% moderate knowledge, and 33.0% low knowledge. Knowledge level was significantly associated with attendance at a recent BLS/CPR workshop (*p* = 0.001) and prior emergency encounter (*p* = 0.001), but not with age, gender, designation, or years of experience (Table 4).

**Table 4:** Association between dentists’ knowledge of BLS/CPR and participant characteristics.

Variables	Knowledge n (%)			*P*-value
	Low 132 (33.0)	Moderate 164 (41.0)	Good 104 (26.0)	
Age				0.304
21–30	72 (37.5)	72 (37.5)	48 (25.0)	
31–40	44 (30.1)	66 (45.2)	36 (24.7)	
≥41	16 (25.8)	26 (41.9)	20 (32.3)	
Gender				0.352
Male	62 (34.8)	76 (42.7)	40 (22.5)	
Female	70 (31.5)	88 (39.6)	64 (28.8)	
Designation				0.284
General dentist	58 (32.6)	80 (44.9)	40 (22.5)	
Intern/house officers	48 (35.8)	52 (38.8)	34 (25.4)	
Faculty/specialist	26 (29.5)	32 (36.4)	30 (34.1)	
Years of experience				0.560
Less than 5 years	52 (38.2)	52 (38.2)	32 (23.5)	
6–10 years	52 (31.3)	68 (41.0)	46 (27.7)	
>10	28 (28.6)	44 (44.9)	26 (26.5)	
Attended BLS/CPR workshop				0.001
Within last 5 years	60 (26.3)	90 (39.5)	78 (34.2)	
Before 5 years	72 (41.9)	74 (43.0)	26 (15.1)	
Encounter an emergency in Clinical practice				0.001
Yes	21 (19.6)	25 (23.4)	61 (57.0)	
No	11 (37.9)	139 (47.4)	43 (14.7)	

**Notes.**

Chi-square test was, *p*-value < 0.05 was considered statistically significant.

Logistic regression analysis was conducted to examine predictors of good knowledge of BLS/CPR among dentists. The overall model was statistically significant (*χ*^2^(9) = 105.6, *p* < 0.001), explaining 34.0% of the variance (Nagelkerke R^2^) with a correct classification rate of 83.5%. The Hosmer–Lemeshow test indicated a good fit (*p* = 0.223). After adjusting for other factors, dentists who had attended a BLS/CPR workshop within the last 5 years were nearly five times more likely to have good knowledge compared to those who attended more than 5 years ago (aOR = 4.91; 95% CI [2.60–9.24]; *p* < 0.001). Similarly, dentists who had previously encountered an emergency were almost 13 times more likely to have good knowledge (aOR = 12.97; 95% CI [6.99–24.06]; *p* < 0.001). Other factors such as age, gender, designation, and years of experience were not significantly associated with knowledge level (Table 5).

**Table 5:** Binary logistic regression predicting good knowledge of BLS/CPR among dentists.

**Predictor Variable**	**OR (Exp B)**	**95% CI**	**p-value**
Age (ref: >41 yrs)			
21–30 yrs	0.61	0.13–2.77	0.520
31–40 yrs	0.46	0.15–1.39	0.168
Gender (ref: Female)	0.61	0.35–1.06	0.079
Designation (ref: Faculty/Specialist)			
General dentist	1.72	0.67–4.42	0.264
Intern/House officer	1.51	0.55–4.10	0.424
Years of experience (ref: >10 yrs)			
<5 yrs	0.69	0.21–2.31	0.547
5–10 yrs	1.12	0.43–2.92	0.812
Attended BLS/CPR workshop (ref: >5 yrs ago)	4.91	2.60–9.24	<0.001
Encountered emergency (ref: No)	12.97	6.99–24.06	<0.001

**Notes.**

Odds ratios (OR) with 95% confidence intervals (CI) are reported, *p*-value <0.05 was considered statistically significant.

Regarding attitudes, the majority of dentists expressed strong agreement with the importance of CPR in dental practice and a moral obligation to intervene during emergencies. However, clinical readiness was suboptimal, with limited availability of emergency protocols and AEDs, and low confidence in performing several key BLS/CPR skills.

Logistic regression was used to identify predictors of confidence in performing CPR. The model was statistically significant (*χ*^2^(9) = 77.96, *p* < 0.001), accounting for 23.9% of the variance (Nagelkerke R^2^), with an overall classification accuracy of 70.0%. The Hosmer–Lemeshow test showed good model fit (*p* = 0.382). Attendance at a BLS/CPR workshop within the last 5 years was the strongest predictor, with dentists nearly seven times more likely to report being very confident compared to those whose training was more than 5 years ago (aOR = 6.93; 95% CI [4.19–11.46]; *p* < 0.001). Male dentists were less likely than females to report confidence (aOR = 0.59; 95% CI [0.37–0.93]; *p* = 0.023). Other factors, including age, designation, years of experience, and previous emergency encounters, were not significantly associated with confidence (Table 6).

**Table 6:** Binary logistic regression predicting confidence in performing CPR among dentists (*n* = 400).

**Predictor variable**	**OR (Exp B)**	**95% CI**	***p*-value**
Age			
21–30 yrs	0.35	0.10–1.23	0.102
31–40 yrs	0.47	0.18–1.20	0.115
Gender (ref: Female)	0.59	0.37–0.93	0.023
Designation (ref: Faculty/Specialist)			
General dentist	2.18	0.99–4.80	0.054
Intern/House officer	1.62	0.70–3.72	0.259
Years of experience (ref: >10 yrs)			
<5 yrs	2.40	0.87–6.61	0.092
5–10 yrs	1.73	0.78–3.83	0.181
Attended BLS/CPR workshop (ref: >5 yrs ago)	6.93	4.19 –11.46	<0.001
Encountered emergency (ref: No)	1.05	0.62–1.76	0.862

**Notes.**

Odds ratios (OR) with 95% confidence intervals (CI) are reported, *p*-value < 0.05 was considered statistically significant.

**Figure 1:**
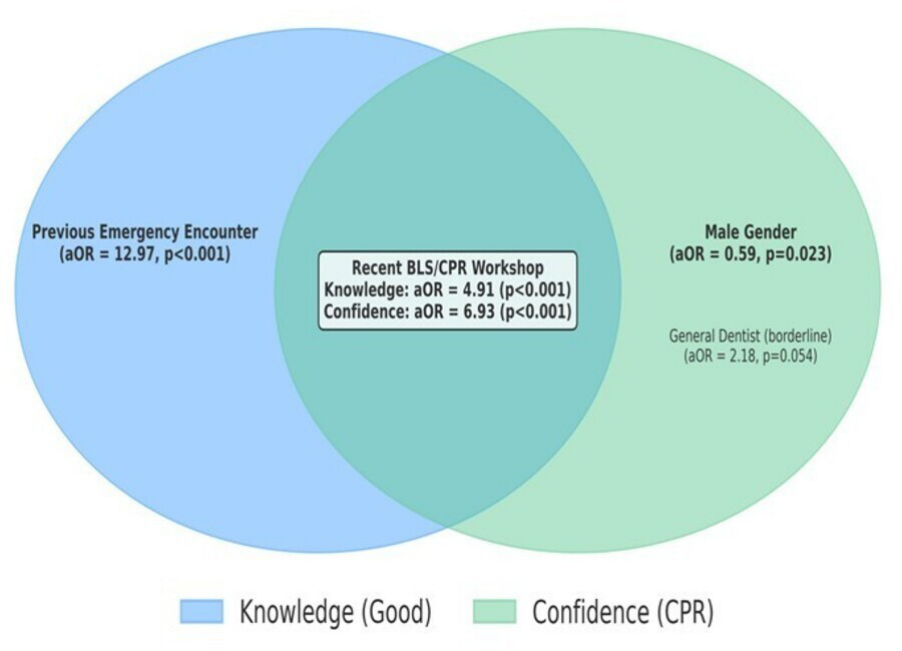
Conceptual model of predictors of good knowledge and confidence in performing CPR among dentists in Saudi Arabia.

Figure 1 illustrates predictors unique to each outcome and those shared across both. Attendance at a recent BLS/CPR workshop was a strong common predictor of both good knowledge (aOR = 4.91, *p* < 0.001) and confidence (aOR = 6.93, *p* < 0.001). Previous emergency encounters were uniquely associated with knowledge (aOR = 12.97, *p* < 0.001), while male gender was negatively associated with confidence (aOR = 0.59, *p* = 0.023). A borderline effect was observed for general dentists being more confident compared to specialists (aOR = 2.18, *p* = 0.054).

## Discussion

Medical emergencies, though relatively uncommon in dental practice, present significant challenges when they occur, requiring immediate and effective intervention to prevent serious outcomes. The present study explored the knowledge, attitudes, and clinical readiness of dentists in Saudi Arabia regarding BLS and CPR. Our findings highlight important gaps between regulatory requirements, self-perceived preparedness, and actual levels of knowledge and confidence.

A key finding was that fewer than one-third of dentists demonstrated good knowledge of BLS/CPR, while the majority displayed only moderate or low levels. This is concerning, given the mandatory requirement of BLS certification for healthcare professionals by the Saudi Commission for Health Specialties (Albaqami & Abozaid, 2019). Similar studies conducted in other Middle Eastern countries have reported comparable deficiencies in dentists’ knowledge and retention of CPR skills, indicating that certification alone may not guarantee competence (Alkandari, Alyahya & Abdulwahab, 2017; Al Ghanam & Khawalde, 2022). This reinforces the well-documented observation that BLS/CPR knowledge and psychomotor skills deteriorate significantly within 6–12 months without regular reinforcement (Wik et al., 2002).

The strong association between recent workshop attendance and knowledge emphasizes the critical role of continuous education. Dentists who attended training within the past five years were nearly five times more likely to demonstrate good knowledge and seven times more likely to report confidence in performing CPR. In Oman, a trial showed that participants who had a short refresher six months after their initial training maintained psychomotor skills substantially better at 12 months compared to those who did not receive refresher training (Al Jadidi & Al Jufaili, 2023). Similar outcomes were reported in Taiwan, where blended training every six months was superior in preserving CPR performance, compared to a 12-month interval group (Chien et al., 2024). In Pakistan, both knowledge and skill retention among bystander trainees declined 6 months post-training unless reinforced (Khan et al., 2022). Experiential learning was also highlighted, as those who had previously encountered medical emergencies were far more knowledgeable than their peers (Khattak et al., 2023). This suggests that exposure to real-life scenarios or simulated drills can play a significant role in reinforcing theoretical knowledge and building confidence.

In terms of attitudes, the overwhelming majority of participants expressed strong agreement with the importance of BLS/CPR in dental practice. Most respondents felt ethically obligated to intervene during emergencies and supported making BLS training mandatory for licensure. These findings reflect a commendable sense of professional responsibility and align with previous studies showing positive attitudes among healthcare providers (Mohammed et al., 2020; Hasnain et al., 2023). However, positive attitudes alone did not translate into adequate preparedness, as knowledge and confidence remained limited. This discrepancy highlights the need to bridge the gap between intention and practice through systemic educational and institutional interventions.

Our study also sheds light on clinical readiness in Saudi dental settings. These findings reflect perceived structural readiness rather than true operational readiness. Less than half of the clinics had established emergency protocols, and only 7.5% reported the presence of an automated external defibrillator (AED). Given that AED use dramatically improves survival in cases of sudden cardiac arrest, this lack of availability is alarming (Chaudhary et al., 2024). Similar deficiencies have been documented globally, in Malaysia, none of the surveyed private dental clinics had an AED (Ramli et al., 2019); and in Poland only about 15.5% of practices reported having one (Smereka et al., 2019). These findings underscore that the lack of AED access is a widespread issue, not confined to any one region. Nonetheless, considering the increasing number of private dental clinics in the Kingdom, ensuring AED availability and functional emergency kits should be a regulatory priority.

Gender differences were another interesting finding. Male dentists were less likely than females to report confidence in performing CPR, despite no significant differences in knowledge levels. Although literature specifically among dental professionals is limited, studies in related medical and educational settings have found gender differences in confidence and self-efficacy. For instance, a recent Saudi study among medical students showed that female students had higher awareness and more favorable attitudes toward CPR than males (Aedh, Abdel-Haliem & Farid, 2025). Previous research among healthcare professionals suggests that gender differences may exist in self-perceived confidence and training engagement. For example, systematic evidence indicates a ‘confidence gap’ in medicine, where female clinicians often report lower self-confidence and self-efficacy compared with male colleagues despite similar objective performance, reflecting differing self-assessments rather than actual competence (Schaffler-Schaden et al., 2023). Additionally, some studies have identified gender differences in willingness and attitudes toward emergency response tasks, including CPR, where factors such as training experience and confidence influence willingness to act (Ok et al., 2025). Therefore, exploring gender-related patterns in confidence and readiness within our sample of dentists aligns with previous findings in health professions and provides context for interpreting our results.

Designation and years of experience were not significant predictors of knowledge or confidence. This finding suggests that seniority or specialization alone does not ensure preparedness, and that ongoing, structured training is necessary for all categories of dentists, from interns to consultants. It also reinforces the importance of aligning continuing professional development (CPD) requirements with evidence-based practices to ensure sustained competency across the workforce.

From an educational perspective, these findings underscore the need for stronger integration of BLS/CPR training into undergraduate and postgraduate dental curricula and ensuring regular, hands-on refresher sessions. Recent workshop attendance strongly predicted both knowledge and confidence, emphasizing that skill retention is time-sensitive. Periodic, practical reinforcement can help maintain competence and readiness, ensuring that training translates into real-world preparedness for managing emergencies in dental practice. Simulation-based training and regular mock drills have been shown to improve retention and performance of emergency skills (Reed et al., 2016). International best practices suggest that short, frequent refresher courses are more effective than long intervals between recertification (Reed et al., 2016). Furthermore, interprofessional training involving dentists, nurses, and other staff may enhance team coordination in real emergency situations.

At the policy level, regulatory authorities should consider mandating periodic re-certification and implementing monitoring mechanisms to ensure compliance in both public and private sectors. Clinics should also be required to maintain functional emergency equipment, including AEDs, and establish standardized protocols for managing emergencies. These measures, combined with educational reforms, would strengthen the overall emergency preparedness of dental professionals and improve patient safety in the Kingdom.

This study has a few limitations. First, its cross-sectional design precludes establishing causal relationships between predictors and outcomes. Second, data were collected using a self-administered questionnaire, which may be subject to response and recall bias, particularly in self-assessment of confidence. Third, the use of a convenience sampling strategy may limit the generalizability of the findings. Additionally, the questionnaire did not capture the geographic region of participants’ practice; therefore, it was not possible to assess regional representation or determine whether the sample was evenly distributed across different regions of Saudi Arabia. As a result, the findings should be interpreted with caution, as they may not fully reflect the national distribution of dentists across the Kingdom. Although internal consistency reliability was assessed, test-retest reliability was not evaluated, and a quantitative content validity index was not calculated. Therefore, the temporal stability and reproducibility of the questionnaire could not be fully established. Future studies should incorporate repeated measurements and formal psychometric validation to further strengthen the assessment tool. Lastly, actual skills in performing CPR were not assessed; observational or simulation-based evaluations would provide a more accurate measure of competence.

Despite these limitations, the study provides valuable insights into the preparedness of dentists in Saudi Arabia regarding BLS/CPR. While attitudes toward CPR are generally positive, knowledge and confidence remain suboptimal, and clinical readiness is hindered by inadequate equipment and protocols. The findings highlight the urgent need for continuous training, curriculum reforms, and institutional policies to strengthen emergency preparedness in dental settings.

### Conclusion

This study demonstrates that while dentists in Saudi Arabia generally exhibit positive attitudes toward BLS/CPR, substantial gaps exist in knowledge, confidence, and clinical readiness. Recent BLS/CPR training and prior exposure to medical emergencies were the strongest predictors of preparedness. These findings highlight the need for regular, hands-on refresher training, stronger curricular integration, and enforcement of clinic-level emergency preparedness measures, including AED availability, to enhance patient safety and professional competence.

## Supplemental Information

10.7717/peerj.21098/supp-1 Supplemental Information 1 Data

10.7717/peerj.21098/supp-2 Supplemental Information 2 STROBE checklist

10.7717/peerj.21098/supp-3 Supplemental Information 3 Questionnaire used for collection of data for this study

10.7717/peerj.21098/supp-4 Supplemental Information 4 Questionnaire codes used in SPSS for data analysis

## Additional Information and Declarations

### Data Availability

The following information was supplied regarding data availability: The raw data is available in the Supplemental File.
