# Supplementary material for: Assessment of clinical readiness, knowledge and attitude, regarding Basic Life Support (BLS) and cardiopulmonary resuscitation (CPR) skill among dentists practicing in Saudi Arabia
Source: PeerJ. 2026 May 6;14:e21098. doi: 10.7717/peerj.21098 (PMC13156951; doi:10.7717/peerj.21098)
Supplement: Supplemental Information 2 [file peerj-14-21098-s002.doc]

**Completed STROBE checklist**

This checklist was elaborated using formal items recommended for cross-sectional studies from STROBE statement (https://www.strobe-statement.org).

|  | Item No | Recommendation | Respected? | Comments and quotes | |
| --- | --- | --- | --- | --- | --- |
| **Title and abstract** | 1 | (*a*) Indicate the study’s design with a commonly used term in the title or the abstract | Yes | | Abstract includes “Cross-Sectional Study”; abstract specifies design. |
|  |  | (*b*) Provide in the abstract an informative and balanced summary of what was done and what was found | Yes | | Abstract clearly states aim, methodology, main results, and conclusions. |
| Introduction | | |  | |  |
| Background/rationale | 2 | Explain the scientific background and rationale for the investigation being reported | Yes | | Background discusses BLS and CPR importance, gaps in practices, and need for standardization. |
| Objectives | 3 | State specific objectives, including any prespecified hypotheses | Yes | | This study aims to assess the knowledge, attitudes, and clinical readiness regarding BLS and CPR among dentists in Saudi Arabia and identify factors associated with preparedness and confidence in managing medical emergencies.. |
| Methods | | |  | |  |
| Study design | 4 | Present key elements of study design early in the paper | Yes | | Identified as a cross-sectional study in Methods. |
| Setting | 5 | Describe the setting, locations, and relevant dates, including periods of recruitment, exposure, follow-up, and data collection | Mostly | | Setting, contexts, dates of inclusion, are fully described in the method section.  Data collected through a validated, self-administered online questionnaire; conducted across dental professionals in Saudi Arabia. |
| Participants | 6 | (*a*) Give the eligibility criteria, and the sources and methods of selection of participants | Yes | | Study population is described is the method section, as well as selection criteria |
| Variables | 7 | Clearly define all outcomes, exposures, predictors, potential confounders, and effect modifiers. Give diagnostic criteria, if applicable | Yes | | Variables include socio-demographics, BLS and CPR knowledge, attitude and readiness, binary logistic regression used to assess predictors of good knowledge. |
| Data sources/ measurement | 8* | For each variable of interest, give sources of data and details of methods of assessment (measurement). Describe comparability of assessment methods if there is more than one group | Yes | | For each variable of interest, data were collected using a structured, validated online questionnaire specifically developed for this study. The questionnaire included sections on:  The questionnaire underwent expert review for content validity and pilot testing, demonstrating good internal consistency. All participants received the same version of the survey, ensuring consistent assessment methods across respondents. |
| Bias | 9 | Describe any efforts to address potential sources of bias | Yes | | We notably tried to reduce bias by excluding incomplete questionnaire. The analysis section also explains this part. |
| Study size | 10 | Explain how the study size was arrived at | Yes | | The method describes the sample size estimation for this study.  It was calculated using the formula for estimating a single population proportion. Assuming a 50% prevalence of adequate CPR knowledge among dentists (to maximize sample size), a 95% confidence level, and a 5% margin of error, the calculated sample size was 400. |
| Quantitative variables | 11 | Explain how quantitative variables were handled in the analyses. If applicable, describe which groupings were chosen and why | Yes | | Quantitative variables, including participant age, gender, designation, years of experience, encounter emergency in dental practice and attended previous BLS/CPR workshop. Descriptive statistics (frequencies, percentages, means, and standard deviations) were used to summarize participant characteristics. |
| Statistical methods | 12 | (*a*) Describe all statistical methods, including those used to control for confounding | Yes | | All statistical analyses were performed using SPSS version 27. Descriptive statistics (frequencies, percentages, means, and standard deviations) were used to summarize participant characteristics. Associations between demographic variables and outcomes (knowledge, attitudes, and readiness) were examined using chi-square tests, t-tests, and ANOVA as appropriate. A p-value <0.05 was considered statistically significant. Binary logistic regression was performed to identify independent predictors of (i) good knowledge of BLS/CPR (good vs. low/moderate) and (ii) confidence in performing CPR (very confident vs. not confident). Independent variables entered into both models included age, gender, designation, years of experience, attendance at a BLS/CPR workshop, and prior emergency encounter. Adjusted odds ratios (aOR) with 95% confidence intervals (CI) were reported. Model fit was evaluated using Omnibus χ², −2 Log Likelihood, Nagelkerke R², Hosmer–Lemeshow test, and classification accuracy. |
|  |  | (*b*) Describe any methods used to examine subgroups and interactions | Yes | |  |
|  |  | (*c*) Explain how missing data were addressed | Yes | | This is described in the method section.  “Missing values were not inferred” |
|  |  | (*d*) If applicable, describe analytical methods taking account of sampling strategy | N/A | | Non applicable |
|  |  | (*e*) Describe any sensitivity analyses | N/A | | Non applicable |
| Results | | |  | |  |
| Participants | 13* | (a) Report numbers of individuals at each stage of study—eg numbers potentially eligible, examined for eligibility, confirmed eligible, included in the study, completing follow-up, and analysed. | Yes | | A total of 400 dental professionals were initially approached to participate in the study. They accessed the online questionnaire, and after screening for eligibility (licensed dental practitioners currently practicing in Saudi Arabia). |
|  |  | (b) Give reasons for non-participation at each stage | Yes | | Non-responders or those refusing consent were excluded (not quantified but mentioned). |
|  |  | (c) Consider use of a flow diagram | N/A | | Use of a flow diagram was not deemed appropriate. |
| Descriptive data | 14* | (a) Give characteristics of study participants (eg demographic, clinical, social) and information on exposures and potential confounders | Yes | | Table 1 shows demographics (age, gender, specialty, experience, training). |
|  |  | (b) Indicate number of participants with missing data for each variable of interest | Yes | | Table 1 shows demographics (age, gender, specialty, experience, training). |
| Outcome data | 15* | Report numbers of outcome events or summary measures | Yes | | Pre-, intra-, and post-operative practices summarized in Tables 2–4; knowledge predictors in Table 8. |
| Main results | 16 | (*a*) Give unadjusted estimates and, if applicable, confounder-adjusted estimates and their precision (eg, 95% confidence interval). Make clear which confounders were adjusted for and why they were included | Yes | | Logistic regression results with AOR, 95% CI, and p-values provided. |
|  |  | (*b*) Report category boundaries when continuous variables were categorized | Yes | | Age, experience, and patients/week grouped in tables. |
|  |  | (*c*) If relevant, consider translating estimates of relative risk into absolute risk for a meaningful time period | N/A | | N/A |
| Other analyses | 17 | Report other analyses done—eg analyses of subgroups and interactions, and sensitivity analyses | Yes | | Post-hoc subgroup analyses described. |
| Discussion | | |  | |  |
| Key results | 18 | Summarise key results with reference to study objectives | Yes | | First paragraph of Discussion summarizes main findings and implications. |
| Limitations | 19 | Discuss limitations of the study, taking into account sources of potential bias or imprecision. Discuss both direction and magnitude of any potential bias | Yes | | Description of limitations such as biases, non-probability sampling, and generalizability limitations noted. |
| Interpretation | 20 | Give a cautious overall interpretation of results considering objectives, limitations, multiplicity of analyses, results from similar studies, and other relevant evidence | Yes | | Findings discussed with references to literature and clinical implications. |
| Generalisability | 21 | Discuss the generalisability (external validity) of the study results | Yes | | Study generalizable described in the discussion section.  Discussed applicability to broader dental settings and need for guidelines. |
| Other information | | |  | |  |
| Funding | 22 | Give the source of funding and the role of the funders for the present study and, if applicable, for the original study on which the present article is based | Yes | | This work was funded by the Deanship of Graduate Studies and Scientific Research at Jouf University under grant No. DGSSR-2024-01-01129. |
|  | | |  | |  |

*Give information separately for exposed and unexposed groups.
