# Supplementary material for: Assessment of clinical readiness, knowledge and attitude, regarding Basic Life Support (BLS) and cardiopulmonary resuscitation (CPR) skill among dentists practicing in Saudi Arabia
Source: PeerJ. 2026 May 6;14:e21098. doi: 10.7717/peerj.21098 (PMC13156951; doi:10.7717/peerj.21098)
Supplement: Supplemental Information 4 [file peerj-14-21098-s004.docx]

**Survey Questionnaire of Assessment of the clinical readiness, knowledge and attitude, regarding BLS and CPR skill among dentists of Saudi Arabia**

Please complete the following questionnaire by placing a Cross in the appropriate box.

**SPSS data code is mentioned along with answer options in brackets ( )**

**Section One: Socio-demographic Data:**

1. **Gender**

Male (1) Female (2)

1. **Age:** Exact age_______

21-30 (1) 31-40 (2)

41 and above (3)

1. **Designation**

General Dentist (1) Intern/House officer (2)

Faculty/specialist (3)

1. **Years of Experience**

Less than 5 years (1) 6 to 10 years (2)

More than 10 years (3)

1. **Attended BLS/CPR workshop**

Within last 5 years (1) Before 5 years (2)

1. **Encounter an emergency in Clinical practice**

Yes (1) No (2)

**Section Two: Knowledge and understanding of BLS and CPR (Tick the correct answer)**

| **Knowledge** |
| --- |
| 1. What does the abbreviation “BLS” stand for?  (a) Basic Life Support  (b) Best Life Services  (c) Basic Lung Support  (d) Basic Life Standards |
| 2. What does the abbreviation “CPR” stand for?  (a) Coronary‑pulmonary resuscitation  (b) Cardiopulmonary resuscitation  (c) Cardiac‑pulse resuscitation  (d) Coronary‑pulmonary regurgitation |
| 3.What is the ideal chest compression rate for adults?  A. 60–80 compressions per minute  B. 80–100 compressions per minute  C. 100–120 compressions per minute  D. More than 130 compressions per minute |
| 4. What is the recommended depth of chest compressions?  A. At least 1 inch (2.5 cm)  B. About 1.5 inches (4 cm)  C. At least 2 inches (5 cm)  D. More than 3 inches (7.5 cm |
| 5. What is the correct compression to ventilation ratio?  A. 15:2  B. 5:1  C. 30:2  D. 20:1 |
| 6. What is the first step in BLS after confirming unresponsiveness?  A. Start chest compressions  B. Call for emergency help/activate EMS  C. Give two rescue breaths  D. Look for an AED |
| 7. When and how should an Automated External Defibrillator AED be used?  A. As soon as it is available; turn it on and follow voice prompts  B. Only after 5 cycles of CPR  C. Only if the person is not breathing but has a pulse  D. After giving two rescue breaths |
| 8. Describe the recovery position.  A. Lying flat on the back  B. On the side with the mouth angled downward  C. Sitting upright  D. Prone with arms under head |
| 9. How long should rescuers perform compressions before switching?  A. Every 5 minutes  B. After 30 compressions  C. Every 2 minutes or sooner if fatigued  D. After each shock |

Each correct answer will be scored as “1” and incorrect as “0,” with a total possible score of 9. The knowledge levelis categorized into Low (1-3), moderate (4-6) and Good (7-9) .

**Section Three: Attitude towards BLS and CPR (Tick the correct answer)**

| **Attitude** | Strongly Disagree (1) | Strongly Agree  (2) |
| --- | --- | --- |
| 1.CPR is a critical skill for all dental professionals. (Strongly Disagree – Strongly Agree) |  |  |
| 2. I feel morally obligated to help during a medical emergency. (Strongly Disagree – Strongly Agree) |  |  |
| 3. BLS training should be mandatory for licensure. (Strongly Disagree – Strongly Agree) |  |  |
| 5. I believe BLS is not relevant in dental settings. (Strongly Disagree – Strongly Agree) |  |  |

**Section Four: Clinical Readiness & Emergency Response Capacity Dentist (Tick the correct answer)**

| **Clinical Readiness & Emergency Response Capacity** | Yes (1) | No (2) |
| --- | --- | --- |
| 1.Do you have a BLS/CPR protocol in your clinic? |  |  |
| 2. Is an emergency kit available in your clinic? |  |  |
| 3. Is an AED available in your clinic? |  |  |
| 4. Are you interested in attending a BLS/CPR refresher training? |  |  |
| 5. Confidence in performing chest compressions |  |  |
| 6. Confidence in using an AED |  |  |
| 7. Confidence in managing choking |  |  |
| 8. Confidence in recognizing cardiac arrest |  |  |
